# Miss Nightingale

**Published:** 1910-08-20

**Authors:** 


### The Hospital

A Journal of

The Medical Sciences and Hospital Administration.

New Series. No. 184, Vol. VII. [No. 12[illegible]6, Vol. XLVIII.] SATURDAY, AUGUST 20, 1910.

### Miss Nightingale.

For the medical and the institutional worlds, as for the world of nursing, there is no event of the week which can take precedence of the death on Saturday last of Miss Florence Nightingale, O.M. It is nearly fifty-six years now since with her band of selected nurses she left England for the Crimea, there to earn that title of "The Lady with the Lamp" with which the genius of a great war correspondent endowed her. The death of her old orderly occurred within a day or two of her own, and the ranks of our Crimean veterans are thinning almost daily; yet there must still be many of her old patients alive to whom her death recalls memories of Scutari, even as to us it suggests the beginnings of the whole splendid fabric of British nursing, and the matchless energy and devotion of its late architect. Feminine heroism is no new thing in Britain; but not even Elizabeth Fry or Flora Macdonald can be said to have established so completely her position in the affections of the people as did the organiser of the Crimean War Nursing Service. To recapitulate the details of her career a volume would be necessary; even as lately as in March 1908 we had the pleasure of chronicling in these pages the bestowal of the honorary freedom of the City of London, an honour which had fallen to but one other woman, the late Baroness Burdett-Coutts. Not long before, the Order of Merit had been conferred upon Miss Nightingale, a distinction which remains unique in that no other woman has ever attained it. Of lesser honours, the tale is a lengthy one.

There is always the danger that the main foundation of her success may be overlooked. Not the most unflinching heroism and devotion to duty, not the best intentions or the most saintly character, would have availed our sick and wounded in the Crimea, had not the "Lady-in-Chief" possessed other qualities of a rarer but even more necessary kind. It was no blind chance that led Mr. Herbert (as he then was) to invite Miss Nightingale to proceed to the front, but the natural result of her years of study and toil. Even before she reached her post a leading newspaper, replying to those who objected to the mission entrusted to her, could justly enumerate such a series of qualifications, such a record of services already done, as convinced everyone of her capacity for the task. It was the long years of steady work already accomplished which marked her out for that appointment, and in a few short months made England ring with her fame. So when her opportunity came to her, Miss Nightingale was able to supervise and superintend her hospitals as no other man or woman could have done. Her genius for organisation, her instinct for training and guiding others, her lofty ideals, all obtained full play because she knew every detail of everyone's share of the work from the top to the bottom. To the public it is the "Lady with the Lamp" and her personal services to the sick and wounded that appeal; to medical men and nurses it is rather the manner in which she was able to inspire efficiency and to create order out of chaos which extort the greatest admiration. Within ten days of her arrival at Scutari she managed, in spite of the jealousy which her advent aroused and the official hostility by which she was perpetually hampered, to set going an impromptu field-kitchen capable of supplying eight hundred men with well-cooked food; and everyone has read of her bold appropriation by force of certain needed stores to which she was forbidden access because they had not been officially inspected. It cannot be too often repeated that not even her genius would have availed much had she not trained herself by years of patient, never-ceasing work which qualified her for the great task she undertook.

Nor is it to be forgotten that Miss Florence Nightingale's reputation rests on a lifetime of labour, not alone on what she accomplished in those far-off days at Scutari. The reform of nursing was a task which had already absorbed much of her attention before that, and was to be ceaselessly her study for very many years after. The campaign made her permanently an invalid; yet physical failure left her but the more active mentally. In 1858 appeared a volume of 560 pages on "The Health, Efficiency, and Hospital Administration of the British Army." Pamphlets, reports, papers, and the supervision of the numerous schools and institutions for the teaching of nursing which owed their origin to her, filled her time even to the closing years of her long life. For Miss Nightingale was never one to be satisfied with results achieved while any more work remained to be done; and even after the establishment of district nursing, and the satisfactory footing on which it was finally placed towards the end of last century, she could still see clearly enough that the problem of midwifery nursing was the next thing to be tackled. Thirty years ago she was earnest in forwarding schemes for the better training of midwives, and twenty years ago she herself organised a health crusade in Buckinghamshire with the object of disseminating some ideas of hygiene among the mass of the population. All this, too, from the couch to which perpetual ill-health had for years confined her. To contemplate such unflagging energy is to understand something—none, perhaps, but those who, like one of our most distinguished field-marshals, were her patients at Scutari can ever grasp the whole—of the veneration she inspired among the troops and among the men and women in England of those times. "To few great reformers," says the *Times*, whose correspondents were foremost in establishing her reputation, furthering her schemes, and assisting her in all her works of mercy, "has the mercy been vouchsafed of seeing within their own lifetime results so striking and so beneficial as those that followed the noble efforts of the 'Lady with the Lamp.'" It is for us to see to it that her work is continued and expanded to meet every claim upon it; for us to learn the practical lesson of which her whole life was one perpetual example, the lesson of personal service and duty done. While the memory of Florence Nightingale remains to animate hospital workers in England, surely something of her spirit must imbue the work of alleviating suffering and distress.

